# Comparison of Aesthetic Quality of the Final Scar in Abdominoplasty with Conventional and Mini Inverted t-Scar

**DOI:** 10.3390/medicina55050142

**Published:** 2019-05-15

**Authors:** Sevgi Kurt Yazar, Merdan Serin

**Affiliations:** Istanbul Training and Research Hospital, Department of Plastic Surgery, Istanbul 34384, Turkey; svgkrt@gmail.com

**Keywords:** abdominoplasty, mini inverted t-scar

## Abstract

*Background and objectives:* Abdominoplasty is one of the most commonly performed cosmetic procedures. The excess skin in the conventional abdominoplasty is transversely excised and a single horizontal scar is formed. The mini inverted t-scar abdominoplasty is a modification of the “Fleur-de-lis” technique and involves the use of a small vertical incision in comparison to the long vertical incision. The aim of this technique is to lower the position of the final abdominal scar instead of addressing the horizontal laxity. In this study, we have compared the aesthetic satisfaction, width and the position of the scar with conventional abdominoplasty and mini inverted t-scar abdominoplasty. *Materials and Methods:* Thirty patients undergoing abdominoplasty and breast reconstruction with transverse rectus abdominis flap (TRAM) and deep inferior epigastric flap (DIEP) were included in the study. In 15 patients, abdominal closure with the conventional transverse scar was performed. In the remaining 15 patients, closure with a mini inverted t-scar was performed. Scar width, scar height and satisfaction scores were evaluated in both groups. *Results:* Scar widths, scar heights and patients’ and as well as surgeons’ satisfaction scores were better in the mini inverted t-scar group than the conventional group. *Conclusions:* The visibility of the vertical scar alone should not be a reason to avoid mini inverse t-scar abdominoplasty. Mini inverted t-scar can be an option to achieve a better hidden high-quality scar.

## 1. Introduction

Abdominoplasty is one of the most commonly performed cosmetic procedures [1,2]. The excess skin in the conventional abdominoplasty is transversely excised leading to a single horizontal closure scar. In some cases, the tension following horizontal closure can be high which can result in a hypertrophic and superiorly displaced scar. “Fleur de lis” abdominoplasty with a vertical scar was defined by Dellon [3], especially for post-bariatric patients with vertical skin excess. Despite the usefulness of the technique, it was not very popular among surgeons because of the fear that it might cause high complication rates. [4]. However, several studies have shown similar complication rates with “Fleur-de-lis” when compared to conventional abdominoplasty with better results in selected patients with vertical skin excess [4].

The mini inverse t-scar abdominoplasty has a similar approach to the “Fleur-de-lis” technique and involves the use of a small vertical incision in comparison to the long vertical incision which is usually defined to up to the level of the xiphoid process [5]. This technique aims to lower the position of the final abdominal scar instead of addressing the horizontal laxity. This method can be useful in selected patients with a risk of superior displacement of the scar. This technique can also be utilized in transverse rectus abdominis flap (TRAM) and deep inferior epigastric flap (DIEP) patients to place the scar at a lower level while eliminating the need for a small or superiorly placed flap. Since the skin tension following the closure is also distributed among the horizontal axis we have predicted that the inverse t-scar technique could result in better scar healing.

In this study, we compare the width and height of the scar and the aesthetic satisfaction of patients and surgeons between conventional abdominoplasty and mini inverted t-scar abdominoplasty.

## 2. Materials and Methods

Thirty patients undergoing abdominoplasty and breast reconstruction with transverse rectus abdominis flap (TRAM) or deep inferior epigastric flap (DIEP) were included in the study. Informed patient consents were obtained prior to surgery. Patients with previous abdominal surgery, systemic diseases, or a body mass index lower than 18 kg/m^2^ or higher than 30 kg/m^2^ were excluded from the study.

Mean patient age was 37 (range: 26–49). In 15 patients abdominal closure with conventional transverse scar was performed. In the remaining 15 patients closure with an inverse t-scar was performed. Mean body mass index (BMI) was 24.3 (range: 20–30) (Table 1).

### Operation Technique

**Figure 1:**
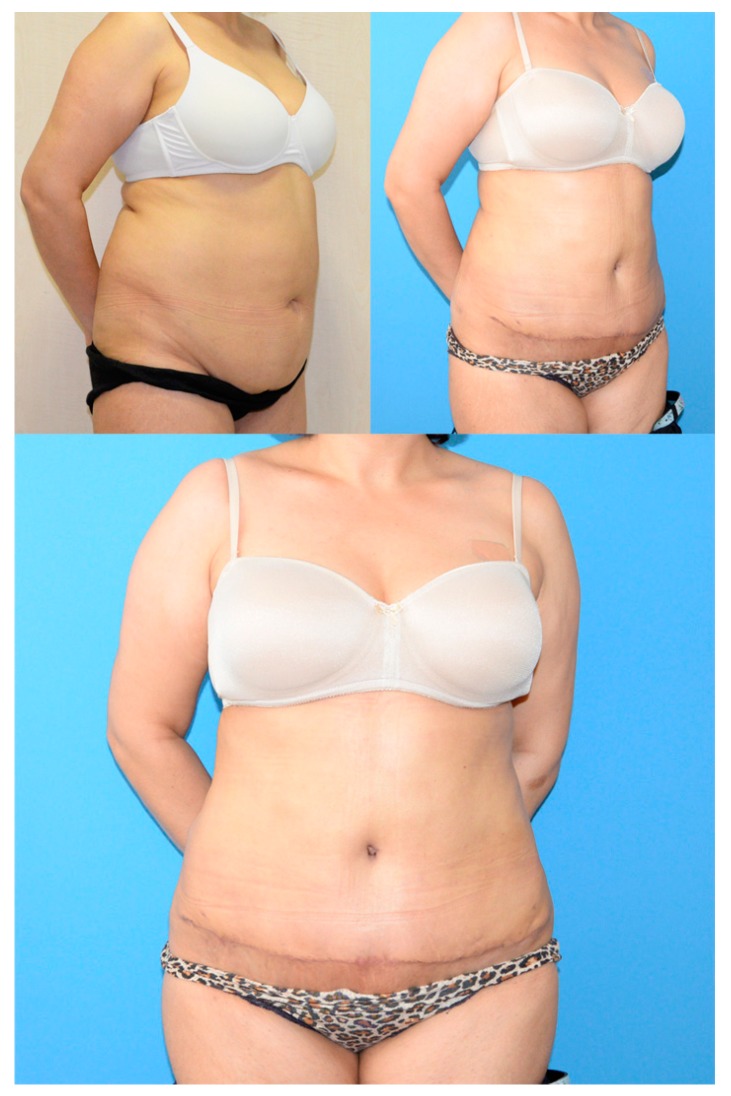
A patient from the conventional horizontal scar group. (**Upper left**) preoperative. (**Upper right**) postoperative oblique view at first year. (**Lower**) postoperative frontal view at first year.

**Figure 2:**
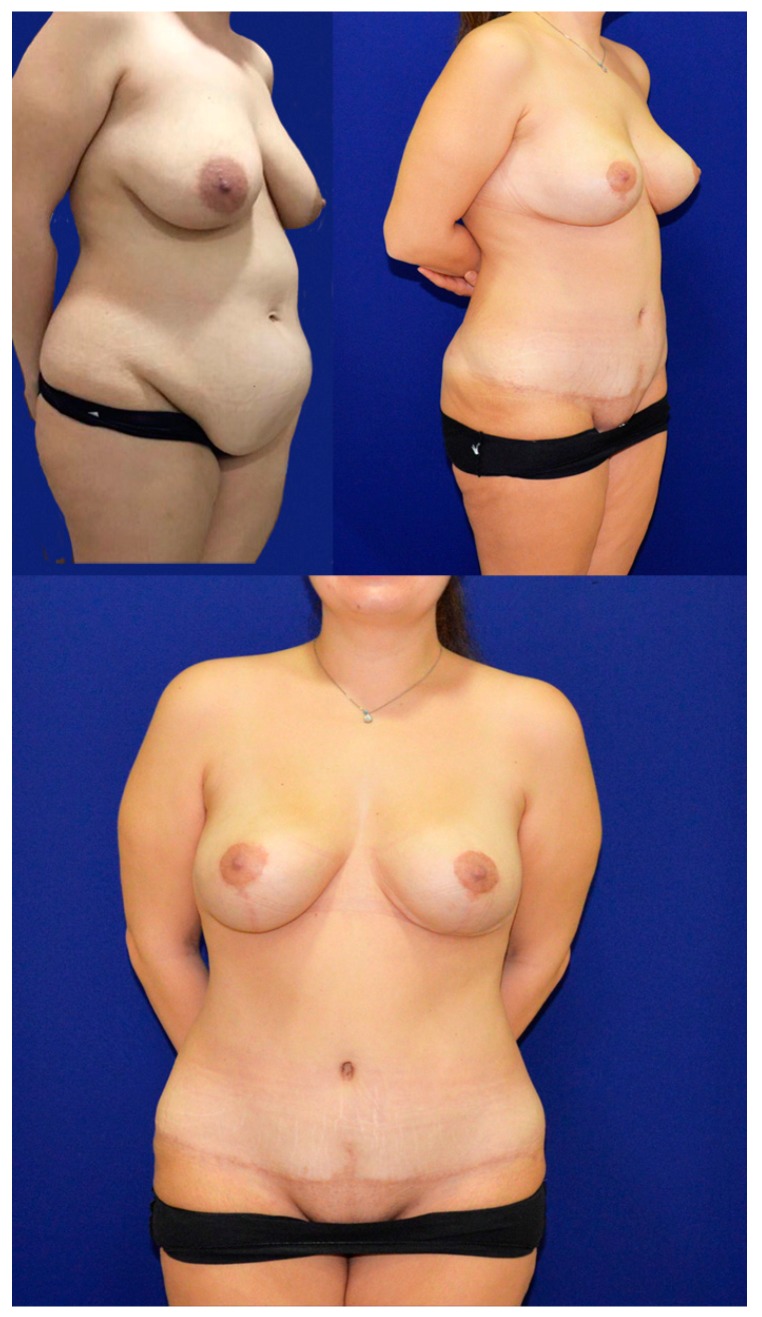
A patient from the inverse t-scar group. (**Upper left**) preoperative. (**Upper right**) postoperative oblique view at first year. (**Lower**) postoperative frontal view at first year.

A low transverse skin incision was made 6–7 cm from the anterior vulvar commissure in the conventional transverse scar group and 8–10 cm from the anterior vulvar commissure in the mini inverted t-scar group. Anterior superior iliac spine was used as a landmark on the lateral edges. The skin flap was dissected while leaving a small amount of loose adipose tissue over the fascia up to the xiphoid process. Umbilicus was separated from the flap with its stalk. The patient was positioned in 30 degrees of flexion, and excess skin was marked and excised. Skin closure in the inverse t-scar group was performed with the addition of a 3–5 cm midline vertical scar to the horizontal scar. Incisions were closed in two layers in both groups. The deeper layer was closed with 2–0 absorbable monofilament sutures and skin layer was closed with 3–0 absorbable monofilament sutures in a subcuticular fashion. Liposuction was performed on all abdominoplasty patients on the flank and anterior abdominal regions. Liposuction was not performed on DIEP/TRAM patients (Figure 1 and Figure 2) (Table 2). None of the patients had major complications such as infection, hematoma, or seroma. Two patients from the conventional scar group and three patients from the inverse t-scar group encountered minor wound healing problems.

**Figure 3:**
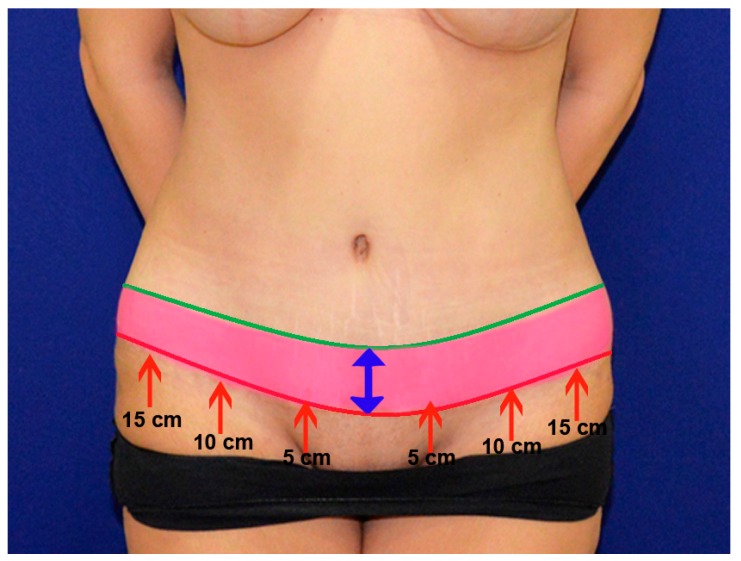
This photo illustrates the potential height difference achieved with the mini inverted t-scar technique (red line) when compared to the conventional technique (green line). The red arrows indicate the points where scar width distance measurements were performed.

Patients were evaluated at postoperative one, three, and six months following surgery. Scars were measured at the postoperative 6-month follow up visit. Scar width was measured at 5, 10, and 15 cm from midline and abdominal incisions, and also at the middle of the vertical incision in inverse t-scar patients (Figure 3). Satisfaction was evaluated by the patient and two independent plastic surgeons who were not involved in the study. The patients and surgeons were asked to rate their scar based on the pigmentation, vascularity, pliability, and height, partially based on the modified Vancouver scar scale [6]. A rating system from 1 to 5 was used (1: worst, 5: best). The distance between the midpoint of the transverse incision scar and the line connecting the anterior superior iliac spines and symphysis pubis was measured to the determine the height of the final scar.

Statistical analysis was performed with Prism 7 software (Version 7.00 for Windows, GraphPad Software, La Jolla California USA). The data was not in normal distribution. A non-parametric Mann–Whitney test was used for comparison of the groups.

## 3. Results

**Figure 4:**
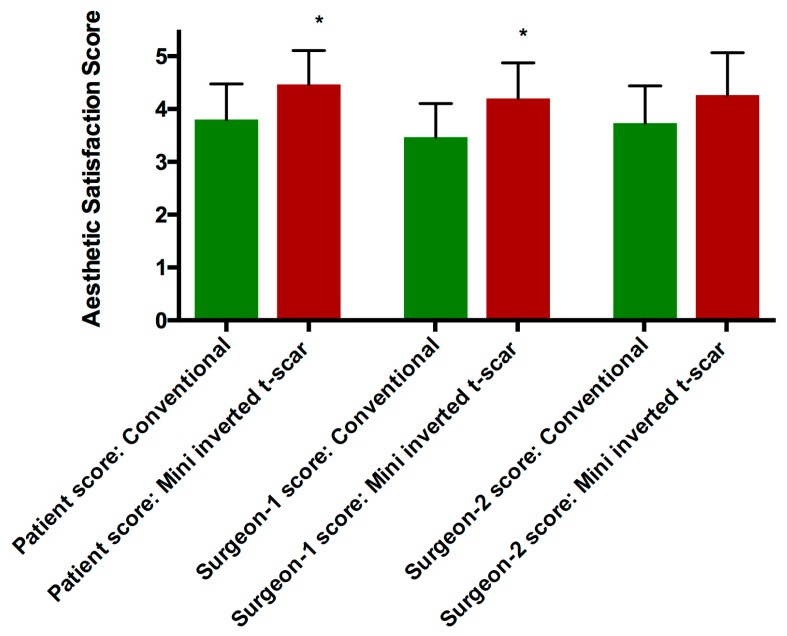
Scar aesthetic satisfaction scores in each group.

The mean of patient aesthetic satisfaction scores were 3.8 ± 0.6 and 4.46 ± 0.6 in the conventional transverse scar and mini inverted t-scar groups, respectively (*p* = 0.0165). The mean of surgeon-1 aesthetic satisfaction scores were 3.46 ± 0.6 and 4.2 ± 0.6 in the conventional transverse scar and mini inverted t-scar groups, respectively (*p* = 0.0081). The mean of surgeon-2 aesthetic satisfaction scores were 3.73 ± 0.7 and 4.26 ± 0.8 in the conventional transverse scar and mini inverted t-scar groups, respectively (*p* = 0.1023) (Figure 4, Table 3).

**Figure 5:**
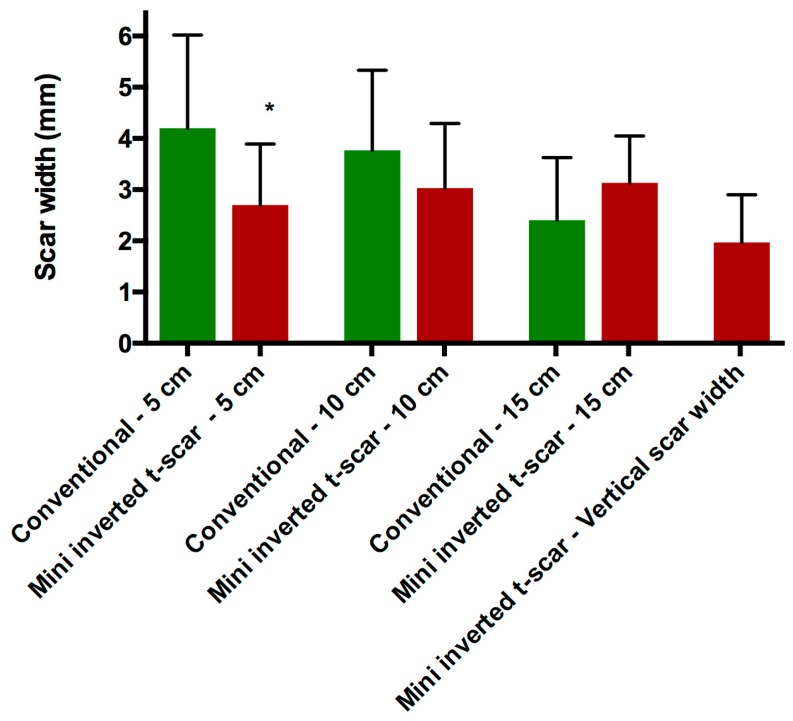
Mean of scar width measurements for each group in mm. (Measurements include 5 cm, 10 cm, and 15 cm from the midline for horizontal scar and single measurement for vertical scar.).

The mean width of the final scar five cm from the midline was 4.2 ± 1.8 mm and 2.7 ± 1.19 mm in the conventional transverse scar and the mini inverted t-scar groups, respectively (*p* = 0.0244). The mean width of the final scar 10 cm from the midline was 3.76 ± 1.56 mm and 3.03 ± 1.26 mm in the conventional transverse scar and the mini inverted t-scar groups, respectively (*p* = 0.2189). The mean width of the final scar 15 cm from the midline was 2.4 ± 1.22 mm and 3.13 ± 0.91 mm in the conventional transverse scar and the mini inverted t-scar groups, respectively (*p* = 0.0553). The mean width of the vertical scar was 1.96 ± 0.93 mm in the mini inverted t-scar group (Figure 5, Table 3).

**Figure 6:**
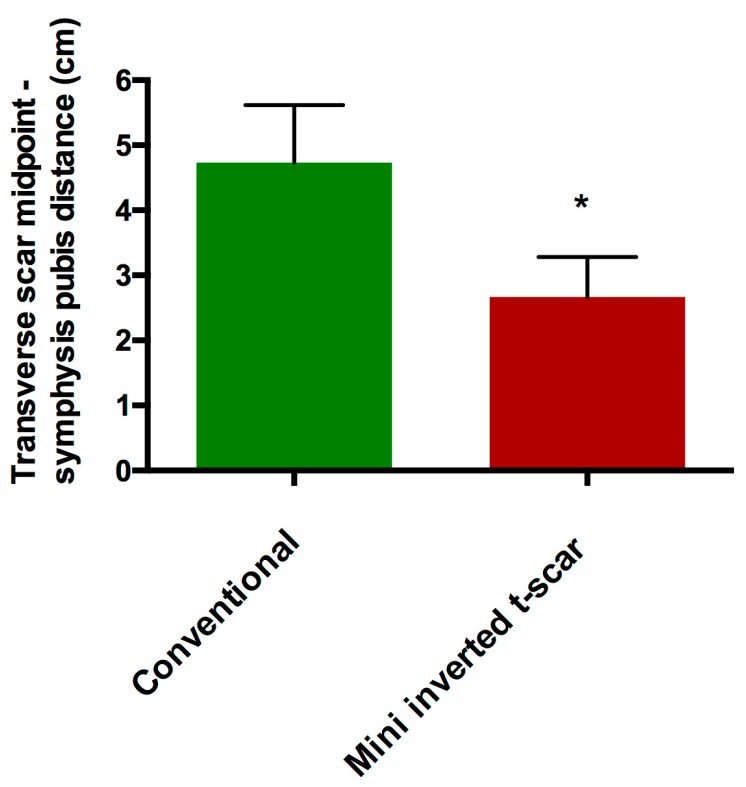
The mean distance from the midpoint of the transverse incision scar to the line connecting the anterior superior iliac spines and symphysis pubis of each group in cm.

The mean distance from the midpoint of the transverse incision scar to the line connecting the anterior superior iliac spines and symphysis pubis was 4.73 ± 0.88 cm and 2.66 ± 0.61 cm in the conventional transverse scar and the mini inverted t-scar groups, respectively (*p* < 0.0001) (Figure 3, Figure 4, Figure 5 and Figure 6, Table 3).

## 4. Discussion

Scar visibility is an important concern in all cosmetic procedures. High tension closure should be avoided to prevent hypertrophic scars [7,8,9]. An ideal scar following a cosmetic surgery should be concealed or should not be easily visible. This is especially true for abdominoplasty which results in a long horizontal scar. The scar should be placed as low as possible to decrease visibility.

Various devices or materials such as scar therapy devices [10], intradermal injection therapies [11], Dermabond [12], and dermal staplers [6] have been utilized to improve the scar quality in abdominoplasties. In order to hide the scar in the lower abdomen the incision is usually planned 6 to 7 cm above the anterior vulvar commissure [13]. However, despite careful planning the incision scar can have a tendency to shift superiorly.

The final height of the abdominoplasty scar depends on the laxity of the mons pubis skin, and the amount of excess skin above the umbilicus. In our experience, in cases with high mons pubis skin laxity or low amount of excess skin, there is a greater risk for superior displacement of the scar. Since these components are highly variable for each patient, it is difficult make a clear definition or to predict the final position of the scar. The addition of the vertical component shifts the position of the transverse scar inferiorly and reduces the tension of the closure. The only disadvantage of this technique is the remaining vertical scar. The length of the vertical scar depends on the amount of skin. In our study, this length was between 3 to 5 cm.

A ‘T’ incision could be used for massive weight loss patient where the skin laxity is present in both the vertical and horizontal dimensions. The ‘T’ incision can also be used in thin patients to address the excess skin. It can also be used to close the original umbilical skin opening that has been pulled down lower.

T-scar closure is not commonly used in abdominoplasty. This is probably due to the concern regarding the visibility of a vertical scar. Due to this concern, inverse t-scar is rarely used in abdominoplasty except in postbariatric patients [3,4,14,15,16,17]. In this study, we have compared the scar quality of conventional horizontal scar abdominoplasties with t-scar abdominoplasties. We have seen that with the conventional horizontal scar technique, the final scar displayed a tendency to displace superiorly despite initial planning. This resulted in a decrease in the distance between the scar and the umbilicus. The mini inverted t-scar technique can reduce the skin tension during the closure and can help position the transverse scar in a lower position on the abdomen.

## 5. Conclusions

In our study, the vertical component of the scar healed very well. This is most probably due to the low amount of tension during the closure. The patients did not complain about the appearance of the vertical scars and did not feel the need to use high waist swimwear following the surgery. In conclusion, the visibility of the vertical scar alone should not be a reason to avoid mini reverse t-scar abdominoplasty. Inverse t-incision can be an option to achieve a less visible scar with an improved aesthetic satisfaction.

## Figures and Tables

**Table 1:** Cohort demographics for patients.

	Conventional (n:15)	Mini inverted t-scar (n:15)
**Mean Age, yr**	37.8 ± 6.7	36.5 ± 7.4
**Age range, yr**	28–48	26–49
**BMI**	25.6 ± 2.9	26 ± 2.9
**BMI range**	20–30	20–30
**Follow-up time (months)**	25.1 ± 8.8	25.5 ± 8.9

**Table 2:** Number of deep inferior epigastric flap (DIEP)/ transverse rectus abdominis flap (TRAM), abdominoplasty, and liposuction patients in each group.

	Conventional (n:15)	Mini inverted t-scar (n:15)
**DIEP/TRAM**	5 (33%)	1 (6%)
**Abdominoplasty**	10 (66%)	14 (93%)
**Liposuction**	10 (66%)	14 (93%)

**Table 3:** Summary of the results. (Mean, standard deviation, and *p* values. * *p* < 0.05).

	Conventional (n:15)	Mini inverted t-scar (n:15)	*p* value
**Patient Aesthetic Satisfaction Score**	3.8 ± 0.6	4.46 ± 0.6	0.0165 *
**Surgeon-1 Aesthetic Satisfaction Score**	3.46 ± 0.6	4.2 ± 0.6	0.0081 *
**Surgeon-1 Aesthetic Satisfaction Score**	3.73 ± 0.7	4.26 ± 0.8	0.1023
**Scar width–5 cm from midline (mm)**	4.2 ± 1.8	2.7 ± 1.19	0.0244 *
**Scar width–10 cm from midline (mm)**	3.76 ± 1.56	3.03 ± 1.26	0.2189
**Scar width–15 cm from midline (mm)**	2.4 ± 1.22	3.13 ± 0.91	0.0553
**Scar width–vertical scar (mm)**	-	1.96 ± 0.93	-
**Distance between midpoint of the transverse scar and the symphysis pubis (cm)**	4.73 ± 0.88	2.66 ± 0.61	<0.0001 *
